# Using neuromorphic computing in prediction of GABA concentration – a pilot study

**DOI:** 10.2478/joeb-2025-0019

**Published:** 2025-12-29

**Authors:** Jie Hou, Abdulkadir Hassen Ali, Ørjan G. Martinsen

**Affiliations:** Department of Physics, University of Oslo, 0316 Oslo, Norway; Department of Clinical and Biomedical Engineering, Oslo University Hospital, 0372 Oslo, Norway

**Keywords:** Neuromorphic computing, GABA, permittivity

## Abstract

Neuromorphic computing has the potential to facilitate detection of GABA concentration levels in the brain, and offers energy-efficient, real-time machine learning processing possibilities. To study whether neuromorphic computing can be used for GABA concentration detection, dielectric relaxation spectroscopy was used to acquire permittivity data of different concentrations of GABA solution. Thereafter, two different machine learning models were compared (Feedforward neural network (FFNN) and convolutional neural network (CNN)) for accuracy in prediction of GABA concentration from dielectric properties. The CNN model was then converted to spiking Neural Networks (SNNs), which showed promising results for energy efficiency and real-time processing capabilities. The system incorporates Tkinter, a Python interface to the Tcl/Tk GUI toolkit for seamless data transfer between the neuromorphic chip and the measurement system, ensuring flexibility and scalability in a user-friendly system.

## Introduction

Accurate measurement of gamma-aminobutyric acid (GABA) is essential for studying the central nervous system and related disorders such as epilepsy, anxiety, and depression. The gold standard for GABA concentration sensing is magnetic resonance spectroscopy (MRS) [1] which is costly and technically complex. The non-electroactive nature of GABA makes detection very difficult – existing chemical and biological detection methods are often invasive, highlighting the need for alternative approaches to GABA concentration sensing [2].

This study utilizes dielectric relaxation spectroscopy (DRS) for permittivity measurement of different GABA concentrations in aqueous solution. The goal of the study is to develop a model that can predict GABA levels. This model will then be integrated into Akida networks, a neuromorphic AI processor platform developed by BrainChip, for real-time interpretation and analysis of the sensor data.

Although DRS provides detailed frequency-dependent measurements, this study evaluates whether hardware-based interpretation using Akida networks offers faster and more efficient analysis by comparing sensor acquisition and processing times. Using hardware for data processing offloads the main processor, improving speed and portability. With further development, the system could monitor GABA levels in vivo and potentially be adapted for clinical use.

Great efforts were made to develop hardware mimicking biological neuronal behavior. Sensors such as e-skin [3], electronic noses, and bio-inspired cameras need real-time, energy-efficient processing. Traditional Von Neumann architecture, with separate memory and processing units, lacks energy efficiency and speed [4] due to constant data transfer.

Neuromorphic systems have gained attention for their potential in spike-based learning, inspired by neural communication. Akida 1000 (BrainChip Holdings Ltd., Sydney, NSW, Australia) is a neural processing device with 1.2 million artificial neurons and 10 billion artificial synapses. The Akida 1000 operates on spikes for inter-neuron communication.

Neuromorphic computing addresses the communication and energy inefficiencies of the Von Neumann architecture by co-locating memory and processing, improving efficiency and speed [5]. The Akida 1000 simulates biological neuron behavior, offering high-speed, low-power AI capabilities. The Akida 1000 trains deep neural networks for tasks like text, audio, and image classification, but published results provide limited quantitative comparisons. While designed for energy efficiency and speed, actual gains depend on the spike-encoding strategy, with time-to-first-spike offering greater benefits than rate coding. Thus, Akida's practical advantages remain implementation-dependent and not fully disclosed.

## Materials and methods

GABA is a vital neurotransmitter in the central nervous system, which regulates neuronal excitability. GABA solutions with concentrations from 10 μM to 200 μM were prepared in deionized water for dielectric relaxation spectroscopy (DRS) measurements. A 0.5 M stock solution was first prepared from ≥99% pure GABA (Sigma-Aldrich) and subsequently diluted in 10 μM increments. All measurements were conducted at a constant temperature of 22 °C, yielding 100 samples across 19 concentrations. Further details can be found in earlier published work [6, 7].

Permittivity measurements were performed using DRS with an open-ended coaxial probe and a vector network analyzer over a frequency range of 200 MHz to 14 GHz. A three-point calibration was performed prior to each measurement session (“Open”, “Short”, and “Load” using deionized water) [8, 9]. A 0.1 M saline solution was used as a verification liquid [10].

### Machine learning model

We used sequential API networks for their simplicity. The multilayer feedforward neural network excels at modeling complex input-output relationships through layers of artificial neurons in a feedforward structure. FFNN and CNN models were investigated in this work. The FFNN, implemented for regression using Keras–TensorFlow, was selected for its ability to model complex relationships in the data. Model architecture and hyperparameters were optimized using KerasTuner with Bayesian optimization. The FFNN adopted a sequential structure comprising 11 layers, using ReLU activation function. The first hidden dense layer featured 382 neurons.

CNNs are specialized neural networks for image and video recognition tasks, leveraging data structure and spatial relationships. They comprise input, convolutional, pooling and dense layers. Training adjusts convolutional layer filter weights to minimize prediction errors, employing techniques like dropout and data augmentation to prevent overfitting.

Our dataset contains 167 data points for each of the four features (dielectric constant, dielectric loss, dielectric conductivity, and loss tangent), can be converted into images. We investigated the use of the 2D convolutional architecture in modeling GABA levels based on the features of the input dataset as an image format.

In this study, we used the Akida 1000 to convert the traditional machine learning model into a Spiking Neural Network operating in the spike domain. This enables real-time learning and predictions at the edge, reducing power consumption and latency for practical applications. Before deploying the architecture, quantization was performed.

### Data quantization

Quantization is used to convert neural network activation and weight values from floating-point format to discrete values, typically represented as events. This conversion involves scaling 32-bit floating-point values to 8-bit unsigned integers within the range of 0 to 255. This scaling causes significant accuracy loss, particularly for variables with decimal values and a large dynamic range such as permittivity data. This precision loss depends on the original value range and scaling factor. Quantization to 8-bit integers is a fundamental requirement in Akida, enabling deterministic spike-based computation while reducing memory usage, power consumption, and data movement by avoiding floating-point arithmetic.

### Akida development environment

The Akida development environment is a machine learning framework specifically designed for modeling spiking neural networks (SNNs). Unlike traditional frameworks like Keras–TensorFlow, Akida focuses on spike-based computation where neurons emit discrete spikes upon reaching predefined firing thresholds. The Akida development environment uses integer-only inputs, outputs, and weights, limiting arithmetic precision. It supports fully connected, convolutional, and separable convolutional layers that combine standard neural network operations with spiking neuron mechanisms, using N-bit weights and 4D input tensors.

Akida supports two main model types: native SNN models and deep learning SNN models. Native SNN models consist of dense layers, with the last fully connected layer being trainable using the Akida edge learning algorithm. In contrast, deep-learning SNN models are CNN models converted to Akida SNN models using the CNN2SNN conversion tool.

Akida models are defined using the sequential API, allowing additional layers to be added. Input layers can be either input data for general data types or input convolutional layers for image-like data structures.

Data-processing layers, including fully connected, convolutional, and separable convolutional layers, follow the input layer. These layers update neuron membrane potentials based on the input and synaptic weights, and use a spiking activation mechanism that triggers a spike only when the membrane potential crosses a defined threshold.

Activation functions use quantization to determine the neuron's response when its potential exceeds the firing threshold. Parameters like threshold, activation bits, and activation step are shared across data-processing layers. Pooling parameters such as pool size, pool type, and pool stride are shared among input convolutional, convolutional, and separable convolutional layers.

### Interfacing system

Tkinter is a Python library for building graphical user interfaces (GUIs). In this study, Tkinter was used to develop a user-friendly system that interfaces with the Akida neuromorphic processor, providing real-time control and access to past measurements. Unlike other GUI frameworks, Tkinter allows for lightweight, cross-platform desktop applications, making it suitable for controlling the processor and managing data storage. The application was rigorously tested for reliability and performance, producing positive results.

This system advancement offers convenient interfacing for tasks like file management and data visualization. The highly customizable Tkinter interface accommodates various applications and operating systems (Windows, macOS, Linux). It efficiently stores and retrieves data, facilitating analysis and training. Its simplicity aids accessibility, while flexibility accommodates diverse measuring systems.

## Results

The final dataset consists of 1900 samples (19 concentrations × 100 samples). Two regression models were built using Keras–TensorFlow's sequential API. Data were normalized and split into 80% training and 20% test sets to ensure balanced feature scaling [11].

**Figure 1:**
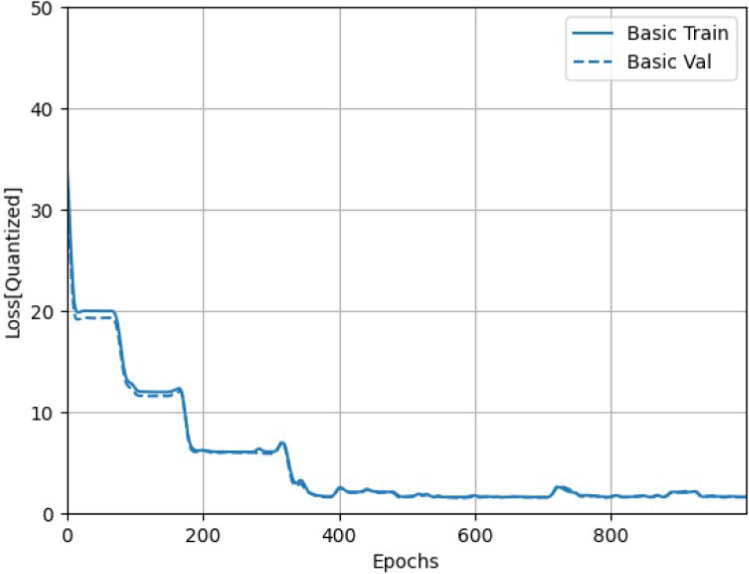
The learning behavior of the Quantized CNN model. From [12].

**Figure 2:**
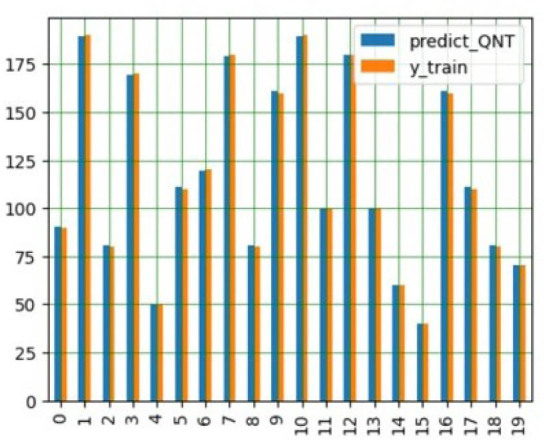
Quantized CNN training and testing prediction compared to the true values. From [12].

We examined both regression models, the FFNN performed well on the GABA dataset but is incompatible with the Akida processor. The CNN was converted into an Akida-compatible spiking neural network through quantization, using 8-bit weights for the first convolutional layer, 2-bit weights for subsequent layers, and 2-bit activations. Results are shown in Figures 1 and 2.

Converting floating-point values to 2D convolutional format for the Akida CNN2SNN poses several challenges. Firstly, this process involves the transformation of floating-point values into an event-based data representation compatible with the Akida CNN2SNN hardware. Achieving this compatibility is intricate and necessitates fine-tuning of the conversion algorithms. Secondly, these conversion algorithms must prioritize low-latency, low-power processing, a critical requirement for the Akida CNN2SNN hardware. Consequently, the accuracy of the results obtained from the Akida CNN2SNN hardware might be comparatively lower than that of traditional floating-point algorithms due to the limited precision of event-based data representations.

Thirdly, the conversion process may elevate the computational complexity of the algorithms, potentially diminishing their performance, especially when adapting them for larger datasets and more complex models. The constraints of event-based data representations and the Akida CNN2SNN hardware architecture can also influence the overall algorithm performance.

Lastly, the Akida CNN2SNN hardware itself may impose restrictions regarding the maximum model size and complexity that it can handle, further constraining algorithm scalability and performance. Our findings highlight the value of applying CNNs and converting them to SNNs on the permittivity dataset to reveal limitations of current neuromorphic hardware.

Table 1 shows the model outcomes, specifically the minimum Mean Absolute Error (MAE) achieved following the training of various algorithms on the dataset over 1000 epochs.

**Table 1:** Model performance on test data.

Model Type	Min MAE	Min MSE	Min MAPE	*R* ^2^	Epochs
ANN	0.8158	2.8870	1.1486	0.9973	1000
CNN (2D)	0.8559	1.3776	0.9822	0.9998	1000
Quantized CNN	1.4768	10.9771	1.8820	0.9962	1000
CNN2SNN	49.4009	N/A	N/A	−0.6259	N/A

## Discussion

The CNN architecture performed well on the regression task while the Akida CNN2SNN exhibited significantly worsened performance due to dataset characteristics and hardware limitations. The Akida platform could only convert CNNs to SNNs and has a 4-bit precision limit for input layers, constraining convolutional analysis of permittivity data across GABA concentrations. These findings underscore the significance of thoughtfully selecting network architecture and optimization methods to attain peak performance in SNNs. Future work should explore the enhanced capabilities of the Akida 2 generation, including higher precision, programmable activations, and support for temporal and event-based neural networks, to better accommodate complex data.

Akida's FPGA-based design limits network size and scalability [13], and the absence of standardized benchmarks complicates performance evaluation. Nevertheless, it remains promising for edge and real-time applications.

To mitigate these constraints, ongoing research is actively engaged in the development of cutting-edge hardware and software tools, alongside the standardization of performance benchmarks and metrics for the evaluation of Akida-based systems. The domain of neuromorphic computing poses a substantial challenge for neophytes, necessitating an in-depth comprehension of various interconnected domains, including neuroscience, nano-electronics, and computer science. Nonetheless, with persistent research and development efforts, the Akida technology holds the potential to ascend as a preeminent neuromorphic computing paradigm, proffering more efficient solutions for real-time processing applications.

Another vital avenue for future research involves the development of a resilient spike encoding mechanism, based on spike count per temporal unit, for representing permittivity data. Rate coding is a well-established information encoding method in the human brain, offering numerous advantages, especially for continuous data. This approach holds the potential to enhance model accuracy and efficiency, empowering them to process substantial volumes of physiological data in real time effectively.

## Conclusion

Our experiment revealed that CNN architecture, optimized structurally, showed the best performance in predicting GABA concentration from permittivity data. Akida's CNN to SNN framework underperformed due to dataset characteristics and Akida's limitations. Despite challenges in applying neuromorphic computing to impedance-based sensory systems, these technologies hold promise for revolutionizing various applications, including real-time processing of extensive physiological data.
